# Electrophysiological Evidence for Interhemispheric Connectivity and Communication in Young Human Infants

**DOI:** 10.3390/brainsci13040647

**Published:** 2023-04-11

**Authors:** Marc H. Bornstein, Clay Mash, Roberto Romero, Amir H. Gandjbakhche, Thien Nguyen

**Affiliations:** 1 Eunice Kennedy Shriver National Institute of Child Health and Human Development, National Institutes of Health, United States Department of Health and Human Services, 8404 Irvington Avenue, Bethesda, MD 20892, USA; clay.mash@nih.gov (C.M.); romeror@mail.nih.gov (R.R.); gandjbaa@mail.nih.gov (A.H.G.); thien.nguyen4@nih.gov (T.N.); 2 Institute for Fiscal Studies, London WC1E 7AE, UK; 3 United Nations Children’s Fund, New York, NY 10017, USA; 4 Environmental Influences on Child Health Outcomes, National Institutes of Health, Bethesda, MD 20852, USA; 5 Department of Obstetrics and Gynecology, University of Michigan, Ann Arbor, MI 48109, USA; 6 Department of Epidemiology and Biostatistics, Michigan State University, East Lansing, MI 48824, USA

**Keywords:** anterior commissure, corpus callosum, human infants, interhemispheric connectivity

## Abstract

Little is known empirically about connectivity and communication between the two hemispheres of the brain in the first year of life, and what theoretical opinion exists appears to be at variance with the meager extant anatomical evidence. To shed initial light on the question of interhemispheric connectivity and communication, this study investigated brain correlates of interhemispheric transmission of information in young human infants. We analyzed EEG data from 12 4-month-olds undergoing a face-related oddball ERP protocol. The activity in the contralateral hemisphere differed between odd-same and odd-difference trials, with the odd-different response being weaker than the response during odd-same trials. The infants’ contralateral hemisphere “recognized” the odd familiar stimulus and “discriminated” the odd-different one. These findings demonstrate connectivity and communication between the two hemispheres of the brain in the first year of life and lead to a better understanding of the functional integrity of the developing human infant brain.

## 1. Introduction

Little is known empirically about connectivity and communication between the two hemispheres of the brain in the first year of life, and what theoretical opinion exists appears to be at variance with the meager extant anatomical evidence. To shed initial light on the question of interhemispheric connectivity and communication, this study investigated brain correlates of interhemispheric transmission of information in young human infants. Research with adults who have had their hemispheres surgically disconnected (i.e., as a “split brain” preparation) has classically indicated that the two hemispheres unequally share mental functions and cannot cross-integrate visual information between their two half-visual fields, so there is little or no perceptual or cognitive interaction between the hemispheres [1,2,3]. For example, commissurotomized patients (who have experienced surgical separation of the two hemispheres of the brain by section of the corpus callosum) almost never make correct same/different judgments (for shapes) presented to a single hemisphere or attend to the left and right visual fields simultaneously and co-ordinate the two [4,5,6,7,8,9,10]. Commissurotomy studies have shown that the two disconnected hemispheres, working on the same task, even process the same sensory information in different ways. Similarly, patients with agenesis of the corpus callosum differ from normals due to a restricted interhemispheric collaboration [11,12]. In brief, the synchronization of neuronal assemblies located in different hemispheres is abolished by the interruption of callosal connections [13,14].

### 1.1. Advantages of Hemispheric Coordination and Its Developmental Meaningfulness

Hemispheric coordination enhances computing capacities relative to unilateral computation as shown by the so-called “bilateral advantage” [15,16,17]. Involvement of both hemispheres (compared to involvement of a single hemisphere only) benefits mental performance by rapid and coordinated engagement of information processing between hemispheres.

In this study, we asked: How early in life are the two hemispheres of the brain in communication? Knowing how early in development interhemispheric connectivity attains functionality, therefore, constitutes a crucial early step toward understanding normal perceptual, language, cognitive, emotional, and social development in humans [18]. The early development of cortico-cortical connectivity is of special interest because the absence of interhemispheric communication would mean that the hemispheres of the infant’s brain are “split”. In early life, this developmental state of affairs would hinder human infants from efficiently integrating functions on the two sides of the body and in the two halves of perceptual space, so the information would remain lateralized to one or the other hemisphere of the infant’s brain. Intact adults enjoy the subjective experience of feeling totally integrated [19], but without interhemispheric communication infants might not. Gazzaniga [2] once opined on neuro-maturational grounds that the normal human infant is born, for all practical purposes, with a split brain in that the two hemispheres do not communicate. Some support for Gazzaniga’s theorizing came from a study showing that the cortically mediated transfer of visual information between the two hemispheres of the brain was not evident before 24 months of age [18].

### 1.2. Commissural Connectivity

Association connections enable communication between cortical regions within the cerebral hemispheres; commissural connections enable communication between corresponding regions in opposite cerebral hemispheres. The right and left hemispheres of the cerebral cortex normally intercommunicate via white matter tracts—the corpus callosum (CC), anterior commissure (AC), and hippocampal commissure [20]. Among these commissural connections, the CC is the largest white matter tract in the mammalian nervous system [21,22]. Fibers of the CC and the AC play principal roles of transferring information between the hemispheres, and their structural integrity subserves perception, attention, memory, language, and reasoning [23,24].

Input to the right hemisphere comes primarily or exclusively from the left hemiretina, and input to the left hemisphere comes primarily or exclusively from the right hemiretina, of each eye. A stimulated region of the cortex sends callosal afferents to symmetrical counterparts of the opposite hemisphere [25], both in terms of connection number (degree) and aggregated weights establishing homotopic commissural connections. That is, mirror-symmetric brain regions share information. Location-specific perceptual learning transfers from a locus in one visual field to a horizontal mirror-symmetric locus in the opposing visual field [26].

When in human development processing in one hemisphere is coordinated with processing in the other hemisphere is the subject of study here. At present, both timing and functionality are poorly understood, as studies of cerebral function in human infancy to date have focused on left vs. right hemispheric specializations and functional hemispheric asymmetries (for example, infants’ right hemisphere advantage for face discrimination) rather than interhemispheric connectivity [27,28,29,30].

Taken together, studies of interhemispheric connectivity and communication in infancy have called on anatomical, electrophysiological, and behavioral evidence. An anatomical study indicated that the CC begins to form at a gestational age of 4 months [31,32], but the CC is among the last brain structures to begin to myelinate and to complete postnatal maturation [33]. Electrophysiological evidence suggests that maturational changes in commissural transmission time correlate with the myelination of the CC [32,34] and has suggested that interhemispheric connectivity is minimal from birth to 2 or 3 years of age [34,35]. Behavioral studies have also been used to assess functional communication between the two hemispheres in infants. To determine whether infants’ left hemisphere (LH) could use information that was learned by the right hemisphere (RH), the transfer of learning from one peripheral visual field to the other was tested in 4- to 10-month-olds [28,29]. Infants learned the task equally well in each visual field, but when tested after their RH had reached a learning criterion, their LH exhibited no facilitation of learning; these results implicated no interhemispheric transfer in infants ages 4–10 months. A subsequent empirical report purported that the cortically mediated transfer of visual information between the hemispheres of the brain is not evident before 2 years of age [18].

In overview, the extant literature on interhemispheric connectivity and communication in infancy is limited by its restricted range of methodology and mixed results. However, anatomical, electrophysiological, and behavioral evidence in infancy may be demonstrative when providing dispositive information, but it may also mislead as it is often insufficiently sensitive, notoriously state-dependent, and subject to many other limitations [36]. Alternative approaches may be more sensitive, appropriate, and revealing. For example, Thomason and colleagues [37] applied resting-state functional MRI to map brain connections in healthy human singleton fetuses between 24 and 38 weeks of gestation. These imaging data confirmed bilateral functional connectivity (especially in the medial and posterior brain regions, notably V1 and the visual association cortices). Connection patterns also increased in strength as the fetuses approached full term. These measurements of the brain’s anatomical structure suggest that the interhemispheric transfer of visual information might yet be demonstrated even in very young postnatal infants. In support of this deduction, infants of 6 to 22 weeks responded differently to familiar and unfamiliar faces presented contralaterally to familiarization input, but only in the right hemisphere [38]. These findings suggest at least a partial transfer of facial image information between the two hemispheres.

### 1.3. The Present Study

Here, we describe a visual evoked potential (VEP) experiment demonstrating the interhemispheric transfer of visual information in young human infants. As cortico-cortical connections between retinotopically organized visual areas are topographically organized, the unilateral stimulation of a visual hemifield projects to the corresponding region in the contralateral hemisphere. The transcallosal transmission of VEP signals generated in one hemisphere in response to a lateralized visual stimulus has previously been recorded over the contralateral hemisphere with a latency suggestive of callosal transmission time [39,40]. Electrophysiological methods could therefore be used to assess connectivity between the hemispheres of the human infant brain. VEPs are relatively easy to obtain in infants, are noninvasive and passive, require little output from participants, and can yield reliable indicators of interhemispheric communication [41]. We studied the interhemispheric processing of visual information early in the first year of postnatal life before speech and language emerged because they could mediate representations of stimuli that infants might otherwise use to succeed at an interhemispheric transfer task.

The present study had the following two goals: first, to determine whether stimulus-dependent changes in cortical synchronized activity can be detected using an electrophysiological technique, and second, to determine whether such a transfer can be ascribed to the activation of cortico-cortical connections. The study used ecologically valid and meaningful stimuli for infants, *viz*. faces [42]. From the earliest moments of life, infants respond to face-like stimuli differently from other stimuli [43,44]. Even in the first hours after birth, infants tend to scan the parts of the face that contain information (usually high-contrast features such as the contours of figures) [45]. Newborns preferentially follow an organized schematic face to a scrambled face [46]; newborns prefer “attractive” faces [47]; and newborns visually recognize their mother’s face based on visual cues alone, even if they have seen the mother for as little as 5.5 h [48,49]. Three-month-olds discriminate within and between facial expressions [50,51]. Cortical electrophysiology indicates that the fusiform face area, a region in the cortex of the human brain specifically dedicated to processing face-related visual input, is already sensitive to faces at 4 months [52]. Five-month-olds discriminate between a face with typical spacing of the eyes and the eyes and mouth, from a face with exaggerated spacing between features [53], and equally young infants are skilled at discriminating other changeable aspects of the face, such as the direction of gaze [54,55].

## 2. Methods

### 2.1. Participants

Prior to beginning this research, all experimental procedures were approved by the Institutional Review Board of the Division of Intramural Research, *Eunice Kennedy Shriver* National Institute of Child Health and Human Development. All methods were carried out in accordance with IRB guidelines and regulations. Infants’ treatment in this study complied with the ethical standards defined by the American Psychological Association and the Society for Research in Child Development and in accord with the Declaration of Helsinki on the ethical treatment of human participants. Parents of all participants were informed and provided written consent prior to the study.

Twelve 4-month-old infants (European Americans, delivered at term, with an appropriate weight for their gestational age and no perinatal complications; *M* age = 126.3 days, *SD* = 11.2; 6 girls) participated. An additional 9 infants participated but provided no usable data due to technical recording failures (*n* = 4) and infant fussiness (*n* = 5). This sample size is consistent with a predominance of infant visual attention studies [56] and is typical of past animal and human infant and adult studies of interhemispheric communication [11,57,58,59,60,61]. Power estimates derived with the present methods indicated adequate power (80%, *α* = 0.05) based on 12 to 14 infants [62].

### 2.2. Procedures

**Figure 1:**
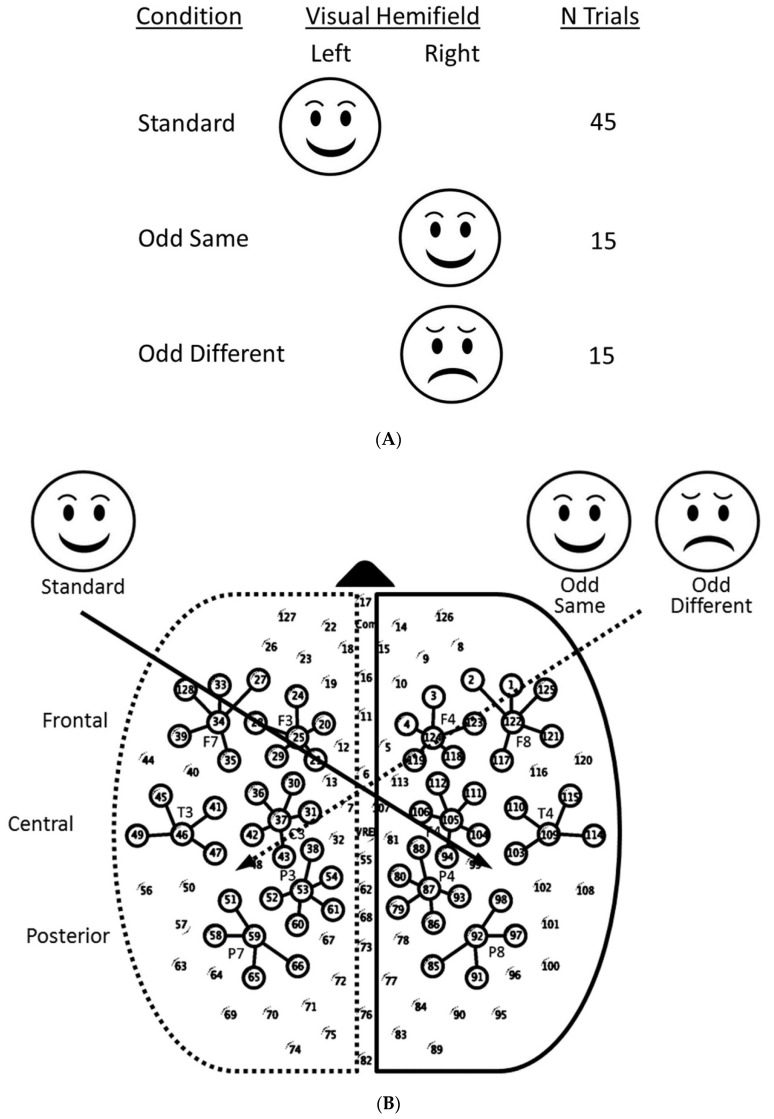
Task design (**A**), sensor layout with lateralized projection of left and right presentation hemifields (**B**).

Infants looked at high-contrast, circular schematic faces, each subtending a 12° diameter and depicted in a white-on-black background. Faces with two different expressions were used: happy and sad (Figure 1A); notably, the faces were composed of the same six elements and differed only in the orientation of three elements, so all stimulus parameters (brightness, number, form, and complexity) between stimuli were equivalent. 

In humans, the left hemiretina of each eye projects to the left cerebral cortex, and the right hemiretina projects to the right cerebral cortex. Anatomical investigations in monkeys have demonstrated that there is a zone of overlap at the retinal vertical meridian (VM), in which ganglion cells projecting to the two cerebral hemispheres intermingle [63,64,65,66], and a study of a human commissurotomized patient confirmed a narrow zone of nasotemporal overlap at the retinal VM, where very limited visual information is encoded by crossed temporal and uncrossed nasal retinal ganglion cells. Bilateral iso-oriented stimuli near the VM, or extending across it, cause an increase in interhemispheric coherence in the EEG. This stimulus-induced increase in coherence disappears after surgical transection of the CC. As the VM around the fovea is represented in both hemispheres, images were presented on either the left or the right of central fixation, and the center of each stimulus was offset left or right of the display center by 10°, resulting in exclusive presentation to the infant’s right or left visual hemifield (Figure 1B). Therefore, the vertical strip of the visual field on each side of the VM was not stimulated.

On each of the 75 randomly ordered trials, infants were presented at 50 cm first with a salient centering animated attention-getter. When the infant was judged to be fixating centrally, the attention-getter was removed, and a stimulus was presented peripherally for 250 ms. The brevity of stimulus exposure ensured that infants could not shift their gaze from central to peripheral to fixate on the peripheral stimulus centrally [11]. Stimulus presentation was followed by a random-duration, inter-trial interval (ITI) during which the screen was uniformly black; ITIs varied in duration from 1800 to 2200 ms. Next, the centering attention-getting animation was re-presented. The stimulus sequence followed a modified oddball paradigm with three trial conditions: one standard same-trial and two different odd-trial conditions. On standard trials, the same stimulus was presented in the same hemifield each time over the course of 45 trials. Randomly interleaved among the standard trials, 30 odd trials presented stimuli in the opposite hemifield. One-half (15) of the trials applied the same stimulus used on standard trials (odd-same), and the other half (15) of the trials applied the novel stimulus (odd different). The facial expression and presentation hemifields, which were used as the standard, were counterbalanced across infants.

### 2.3. EEG Recording

Infant EEGs were recorded with an EGI 128-channel EEG recording system. The EEG signal was referenced to the vertex and recorded with 20 K amplification, at a sampling rate of 250 Hz, with bandpass filters set at 0.1–100 Hz and 40 Ω impedance. Recordings were digitally filtered with 0.4 Hz high-pass and 40 Hz low-pass filters and segmented into standard, odd-same, and odd-different trials. The data were referenced to the average of all channels, and a baseline correction was applied to a 100 ms pre-stimulus recording interval. Recordings were segmented to 1000 ms following the onset of the stimulus. Segments were inspected for artifacts, defined as signal amplitudes exceeding 200 μV or a differential amplitude exceeding 100 μV. A trial was excluded if more than 20% of the channels exceeded these thresholds. Infants were required to have at least 10 artifact-free trials per trial condition to be included in the study. The mean number of trials completed that were free of gross artifacts was 32.5 for standard trials and 24.3 for odd-same and odd-different trials.

The segmented ERP waveforms were preprocessed by using EEGLAB [67] running in Matlab v7.1 (MathWorks, 5404 Wisconsin Ave. Chevy Chase, MD 20815, USA). An independent components analysis was conducted to decompose the signal into separate information sources. An algorithm, an automatic EEG artifact detection based on the joint use of spatial and temporal features (ADJUST) [68], was used to identify and remove components corresponding to four classes of source artifacts: eye blink, vertical eye movement, horizontal eye movement, and generic discontinuity. For each participant, cleaned data were averaged over channels within site clusters and then averaged over trials by the stimulus condition. Grand averages were inspected for the presence of discrete components.

Channels selected for analysis corresponded to a subset of lateralized international 10-10 system sites utilizing target sensors. At each site, additional sensors surrounding the individual target channels were clustered for averaging. For each infant, waveforms were averaged over channels within site clusters (Figure 1B) and then averaged over trials by the trial condition. Grand averages were inspected for the presence of discrete components, and negative deflections were identified between 400 and 800 ms. This Nc component (typically deflecting more negatively in response to odd than standard trials) has been observed in previous studies of infants’ responses in an oddball paradigm [69]. Response amplitude was averaged over the 400–800 ms time window by condition for each infant.

## 3. Results

**Figure 2:**
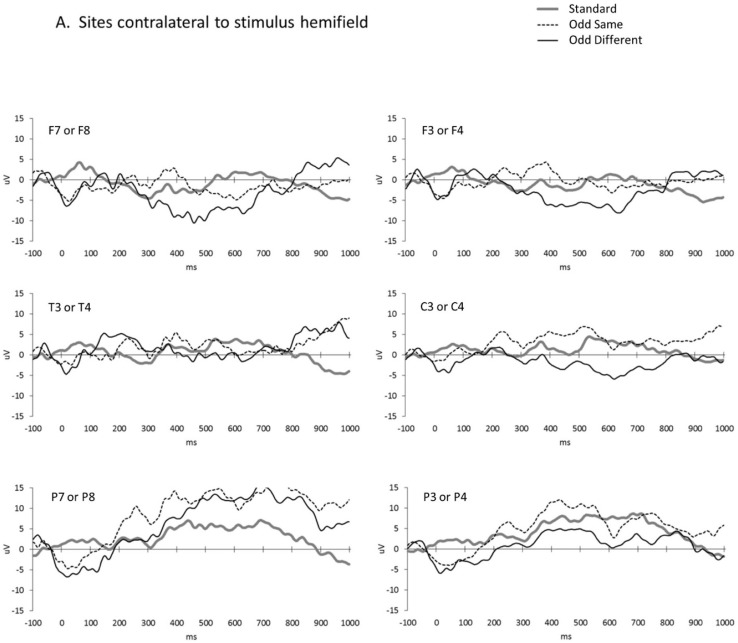

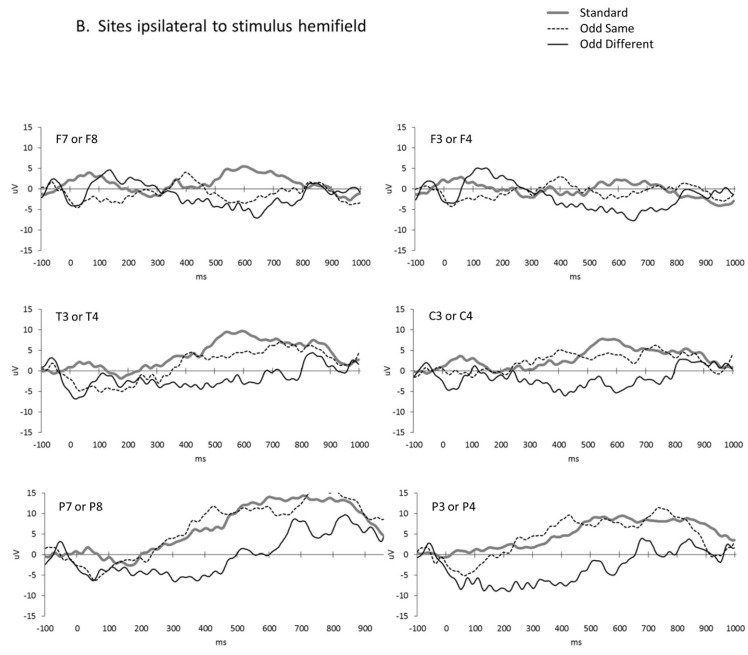
Average waveforms by condition, scalp recording site (e.g., F7 or F8), and stimulus presentation hemifields: (**A**) contralateral and (**B**) ipsilateral.

Figure 2 presents the averaged waveforms by the condition, site, and stimulus presentation hemifields. Because responses are compared between the stimuli and presentation hemifields, it was first necessary to ensure that there were no baseline differences in responses between the stimuli and hemifields. At baseline, infants’ responses did not differ between the stimuli (*F*(1,8) = 0.39, *ns*) or between the presentation hemifields (*F*(1,8) = 0.04, *ns*). Although expressions of complex natural facial stimuli can modulate social attention [70,71], the lack of a baseline difference for these schematic forms indicates a response equivalence in the present context.

**Figure 3:**
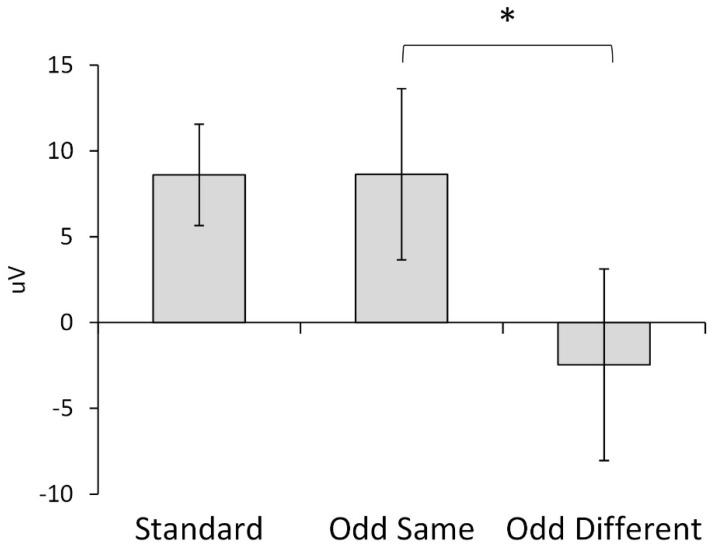
Mean response amplitude showing difference between odd-same and odd-different conditions (* *p* < 0.05).

The main analysis included all of the trials and consisted of an ANOVA incorporating three within-subject factors: the trial condition (planned contrasts of standard vs. odd-same and odd-same vs. odd-different), brain hemisphere (ipsilateral to the presentation hemifield vs. contralateral), and scalp site (frontal vs. central vs. posterior). Three of the site contrasts were significant, with frontal sites having smaller amplitudes than both central (*F*(1,11) = 8.33, *p* = 0.015, η_p_^2^ = 0.431) and posterior (*F*(1,11) = 20.41, *p* = 0.001, η_p_^2^ = 0.650) sites and central sites having smaller amplitudes than the posterior sites (*F*(1,11) = 18.40, *p* = 0.001, η_p_^2^ = 0.626). The trial condition contrast between the standard and odd-same trials was not significant (*F*(1,11) = 0.00, *p* = 0.996, η_p_^2^ = 0.000), but the amplitude of the response to the odd-different trials peaked negatively and differed significantly from the amplitude to the odd-same trials (*F*(1,11) = 8.34, *p* = 0.015, η_p_^2^ = 0.431). Figure 3 shows the significant interhemispheric transfer effect.

The latency to peak amplitude of the response components was also examined by the trial condition and hemisphere. The latencies did not differ between the standard (*M* = 602.12 ms, *SD* = 20.48), odd-same (*M* = 580.04, *SD* = 23.96), and odd-different conditions (*M* = 609.16, *SD* = 23.64) (*F*(2,10) = 0.48, *p* = 0.629), or between the contralateral (*M* = 597.68, *SD* = 13.24) and ipsilateral hemispheres (*M* = 595.48, *SD* = 19.04) (*F*(1,11) = 0.02, *p* = 0.889). No interactions were significant.

## 4. Discussion

This is one of the first studies to provide electrophysiological evidence of interhemispheric functional cortico-cortical connectivity and communication in early human infancy and the first to show them bi-directionally. In humans, callosal fibers synchronize the activity between the two hemispheres [72]. The present study identified synchronized activity of cortico-cortical connections using EEGs. Among the many possible cortico-cortical connections, callosal connectivity appears to provide several advantages. First, as a rule, symmetrical points of the two hemispheres are directly interconnected by callosal axons, and symmetrical cortical areas coactivate during stimulation. On this basis, we hypothesized that, if the EEG conveys activity of cortico-cortical connectivity and communication, then an increase in the EEG in the contralateral cortical locus should be found when one hemifield is stimulated with an identical stimulation presented in the other hemifield across the VM. Stimulus-specific interhemispheric communication is absent in agenesis of the corpus callosum or when the corpus callosum is transected, supporting the hypothesis that information transfer is due to the activation of callosal connections. Callosal transection decorrelates EEG activity between the two hemispheres [73].

The principal anatomical structures known to mediate interhemispheric connectivity and communication in adults are the CC and the AC. The AC is a bundle of nerve fibers connecting the two temporal lobes of the cerebral hemispheres across the midline. The functionality of the AC is still not completely understood; however, the intact human AC appears to transfer no visual information [10,73]. Rather, the great majority of fibers connecting the two hemispheres travel through the CC, which is more than 10 times larger than the AC, and the interhemispheric transfer of visual information points to involvement of CC pathways [74,75,76,77,78,79,80]. Our functional data, therefore, agree with and advance previous observations of fetal interhemispheric connectivity attained by means of anatomical and structural imaging [37,77].

Most fibers in the CC connect homologous regions in the two hemispheres. The connectivity between the same anatomical areas in opposite hemispheres likely subserves mature sensorimotor and cognitive processes that require hemispheric integration. As the neuroanatomical infrastructure matures, one would expect symmetry in bilateral signals to increase, resulting in signaling efficiency. Thomason et al. [37] found bilateral connectivity in one-half of 42 areas tested in the brains of human fetuses, and the strength of connectivity between homologous cortical brain regions increased with advancing fetal gestational age to term. Evoked potentials in the cat visual cortex increase in amplitude if preceded by stimulation of the homotopic area in the contralateral hemisphere [81], and neurons in superficial cortical layers show synchronous excitation to contralaterally evoked callosal potentials, but this effect disappears after section of the CC [82].

The CC’s major subdivisions are organized into functional zones. Sub-regions of the CC (the genu, body, isthmus, and splenium) are topographically arrayed to carry interhemispheric fibers representing heteromodal and unimodal cortical brain regions. Posterior regions, where the response amplitudes were the greatest, are associated with visual information processing [75,76]; fibers crossing through this sub-area are usually small-diameter axons that transfer information between the hemispheres and facilitate higher-order processing in the parietal, temporal, and occipital lobes [83]. Neurological studies have revealed that, when the splenium (a posterior area of the CC that interconnects the occipital lobes) is spared, there is normal transfer of visual information between the two hemispheres [19]. Callosal connections between the occipital cortices in humans have been studied with the Weigert-Pal method in patient cases diagnosed with occipital lesions [84]. Occipital cortices on either side are interconnected via the splenium of the CC. In the present study, we observed the activity of neural circuits underlying posterior scalp electrodes, pointing to the visual cortex as the main pathway of interhemispheric cross-talk. In human infants, callosal fibers connect both the primary and secondary visual areas [85,86].

### 4.1. Limitations

This study makes several contributions to the literatures on brain development, infancy, and visual perception. The study also has certain limitations; here, we mention three. The maturation of sensory systems tends to occur peripherally (at the sense organ) before it occurs centrally (in the brain). For example, the eye differentiates structurally and reaches functional maturity before the visual cortex does [87]. By the second trimester of gestation, however, the eye and visual system are essentially mature structurally, although their levels of functional competence lag behind [88], as vision is the least developed sense at birth. The auditory system is more advanced in development before birth. It could be, therefore, that studies of other sensory systems, such as audition, would lend themselves to stronger interhemispheric (temporal lobe) connectivity and communication. Future studies of interhemispheric cross-talk should investigate and compare these sensory systems.

We used face stimuli in this study. Faces enjoy an advantage relative to other objects and stimuli in visual processing due to either an innate neurological mechanism or quickly learned expertise [89,90]. Lettvin et al. [91] proposed the term “grandmother cell” to describe individual neurons that respond best to hypercomplex stimuli, such as faces [92]. EEG responses of 3-month-old infants differ between familiar and unfamiliar faces at different sites on the scalp, corresponding topographically to what has been observed in adults [93,94]. It could be that the interhemispheric connectivity and communication demonstrated in this study are specific to face visual stimuli. In consequence, other visual stimuli should be tested in future research.

Finally, we averaged over all of the EEG sites to demonstrate the interhemispheric transfer but collected data from several sites and over time (Figure 2 and Figure 3). It could be that larger interhemispheric transfer effects occur at certain sites or at certain lags. For example, the temporal sites (T3 or T4, especially ipsilateral to the stimulus hemifield) seem to show large effects across an extended duration. The temporal gyrus is known to play a role in multisensory tasks (e.g., reading), so individual differences in infant and child maturation of the corpus callosum could impact temporal gyrus multisensory integration competencies [95]. Future extensive and complex time x sites x hemifield analyses with infants and children of different ages would be necessary to shed light on this dynamic.

### 4.2. Implications

The results of this study improve our understanding of central nervous system development in early life. The results also speak to fetal and infant functional brain development, which is linked to cognitive and mental health outcomes [96]. For example, morphological abnormalities of the CC are associated with degraded behavioral development and cognitive processes in primates, including humans [97,98]. The absence of interhemispheric connectivity is linked to pervasive cognitive limitations [98,99]. Additionally, a substantial clinical literature attests that many developmental and psychiatric disorders are attributable to disruptions or alterations in the neural or functional connectivity of brain networks, such as autism spectrum disorder, attention deficit hyperactivity disorder, schizophrenia, depression, and posttraumatic stress [14,42,98,99,100,101,102,103,104,105]. Abnormal patterns of brain connections likely originate early in life, during in utero fetal or infant development, and have consequences for later health development in the lifespan.

Interhemispheric connectivity and communication play pervasive roles in normal perceptual, cognitive, verbal, and socio-emotional development. On this account, the once-theorized split-brain neurological state of the normal infant [2] would present startling impediments to development at the start of life. Rather, normative functional interhemispheric connectivity and communication (as demonstrated here) likely begins in infancy or before (as demonstrated in [37]). Our design offers a new noninvasive method to examine functional connectivity and communication of interhemispheric networks during human infancy and extends the possibility of clinical diagnosis of early life insults to the brain, independent of the sensory, motor, and cognitive abilities of the patient, for the subsequent development of disorders.

## 5. Conclusions

Considering the multiple dependencies of the brain on interhemispheric communication, a better understanding of the early development of cerebral connectivity is crucial. To date, studies of functional connectivity and communication have rested mainly on limited anatomical and structural or equivocal electrophysiological and behavioral data. The present study used VEPs to sensitively test for the interhemispheric transfer of visual information in 4-month-old infants. The equivalent brain response between standard and contralateral odd-same trials, and distinguishing brain responses between ipsilateral odd-same and odd-different trials, indicated recognition of the odd-same and discrimination of the odd-different stimuli in relation to the contralateral standard, even though the stimulus input was restricted hemispherically between the odd and standard trials. The observed distal responses are likely attributable to the spread of activation from the stimulation site in one hemisphere along the corpus callosum anatomical pathways between the hemispheres to the analogous site in the other hemisphere. These findings provide the first electrophysiological evidence of interhemispheric connectivity and communication in early infancy, and the technique opens up new vistas for in vivo studies of neural connectivity and the interhemispheric transfer of visual information in the young human infant brain. Additionally, this technique holds potential for the clinical study of disorders that depend on functional interhemispheric connectivity.

## Data Availability

The study data can be made available to authorized parties upon application to the corresponding author.

## Abbreviations

anterior commissure (AC), corpus callosum (CC), magnetic resonance imaging (MRI), left hemisphere (LH), right hemisphere (RH), vertical meridian (VM), visual evoked potential (VEP).
